# Clinical effects of direct hemoperfusion using a polymyxin B-immobilized fiber column in clinically amyopathic dermatomyositis-associated rapidly progressive interstitial pneumonias

**DOI:** 10.1186/s12890-017-0479-2

**Published:** 2017-10-24

**Authors:** Hiroko Okabayashi, Hidenori Ichiyasu, Sayuri Hirooka, Kimitaka Akaike, Keisuke Kojima, Takayuki Jodai, Yasumiko Sakamoto, Hideharu Ideguchi, Shohei Hamada, Chieko Yoshida, Susumu Hirosako, Shinichiro Okamoto, Hirotsugu Kohrogi

**Affiliations:** Department of Respiratory Medicine, Kumamoto University Hospital, Faculty of Life Sciences, Kumamoto University, 1-1-1 Honjo, Chuo-ku, Kumamoto, 860-8556 Japan

**Keywords:** Dermatomyositis, Clinically amyopathic dermatomyositis, Rapidly progressive interstitial pneumonias, Polymyxin-B direct hemoperfusion

## Abstract

**Background:**

Rapidly progressive interstitial pneumonias (RPIPs) associated with clinically amyopathic dermatomyositis (CADM) are highly resistant to therapy and have a poor prognosis. Multimodal therapies, including direct hemoperfusion using a polymyxin B-immobilized fiber column (PMX-DHP), have a protective effect on RPIPs. We evaluated the effects of PMX-DHP on CADM-associated RPIPs.

**Methods:**

We retrospectively enrolled 14 patients with CADM-associated RPIPs and acute respiratory failure treated with PMX-DHP, corticosteroids, and immunosuppressive agents. Clinical manifestations were compared between survivors and non-survivors at 90 days after PMX-DHP.

**Results:**

The survival rate at 90 days after PMX-DHP was 35.7% (5/14). Before PMX-DHP, the survivor group exhibited a significantly higher PaO_2_/FiO_2_ (P/F) ratio and serum surfactant protein-D (SP-D) levels and significantly lower lactate dehydrogenase (LDH) and ferritin levels than the non-survivor group. Platelet counts were significantly decreased after PMX-DHP therapy in both groups, but remained higher in the survivor group than the non-survivor group over the course of treatment. Anti-melanoma differentiation-associated gene 5 (MDA-5) antibody positive patients demonstrated a poor 90-day survival rate, lower platelet counts and P/F ratio, and higher LDH levels than anti-MDA-5 antibody negative patients.

**Conclusions:**

CADM-associated RPIPs with anti-MDA-5 antibody is associated with a very poor prognosis. A higher P/F ratio and SP-D level, lower LDH and ferritin levels, higher platelet counts, and anti-MDA-5 antibody negativity are important prognostic markers in patients with CADM-associated RPIPs treated with PMX-DHP.

## Background

Dermatomyositis (DM) is an autoimmune disorder affecting the muscles, skin, joints, heart, and lungs. Interstitial pneumonia arises in approximately 40%–50% of patients with DM. The clinical course of interstitial pneumonia with DM varies. Rapidly progressive interstitial pneumonias (RPIPs) are a serious complication of interstitial pneumonia with DM and a leading cause of death among patients with the condition [1]. Clinically amyopathic DM (CADM) is characterized by typical dermatologic findings of DM with no or minimal muscle weakness. As reported extensively in Asia, patients with CADM often develop RPIPs with acute respiratory failure [2]. CADM-associated RPIPs are resistant to combination therapy with high-dose corticosteroids and immunosuppressive agents, and have a poor prognosis. Recently, the anti-melanoma differentiation-associated gene 5 (MDA-5) antibody was demonstrated to be involved in DM and reflect the response to treatment and outcome of CADM-associated RPIPs [3–5]. The anti-MDA-5 antibody is mutually exclusive to anti-aminoacyl-tRNA synthetase (ARS) antibodies, a group of representative antibodies detected in DM and polymyositis (PM).

Direct hemoperfusion therapy using a polymyxin B-immobilized fiber column (PMX-DHP) (Toraymyxin®; Toray Medical Co., Tokyo, Japan) as an extracorporeal blood filter was developed to remove endotoxin from the blood and is used for the treatment of patients with endotoxemia and septic shock [6–8]. PMX-DHP therapy improves hemodynamic status and respiratory dysfunction in patients with septic acute respiratory distress syndrome (ARDS) [9–11]. Furthermore, PMX-DHP adsorbs and eliminates excessive inflammatory cytokines and mediators and activated leukocytes, which are essential for the pathogenesis of ARDS.

Recently, several studies have reported the protective effects of multimodal therapies involving PMX-DHP on RPIPs such as acute exacerbations of idiopathic pulmonary fibrosis (IPF) or fatal drug-induced interstitial pneumonias in which endotoxin is not detected in the blood [12–16]. In a larger, retrospective multi-center study of PMX-DHP in patients with RPIPs, including acute exacerbation of IPF, Abe et al. reported a favorable outcome; however, this study did not include a control group without PMX-DHP [15]. Recently, the results of several retrospective studies including ours conducted to comparatively analyze PMX-DHP and control groups revealed significantly improved outcomes with PMX-DHP therapy [16, 17]. Abe et al. [18, 19] demonstrated that PMX-DHP treatment eliminated activated neutrophils and humoral factors, including matrix metalloproteinase-9 (MMP-9) and high-mobility group box protein 1 (HMGB-1), from the blood circulation of patients with acute exacerbations of IPF. These factors contribute to acute lung inflammation by inducing the accumulation of neutrophils and production of proinflammatory cytokines in the lung [20], both of which are relevant to an improvement of the P/F ratio.

Additionally, PMX-DHP has been employed in the treatment of aggressive interstitial pneumonia due to CADM [21–24]. We reported the cases of three patients with CADM-associated RPIPs treated with PMX-DHP in combination with conventional therapy, in whom the PaO_2_/FiO_2_ (P/F) ratio, serum lactate dehydrogenase (LDH) levels, Krebs von den Lungen-6 (KL-6) levels, and abnormal shadows on chest high-resolution computed tomography (HRCT) improved and all of whom survived [21]. However, the efficacy of additional PMX-DHP for the treatment of CADM-associated RPIPs remains unclear. In this study, we investigated the clinical effects of PMX-DHP on CADM-associated RPIPs receiving conventional therapy.

## Methods

### Patients

We retrospectively enrolled 14 patients with CADM-associated RPIPs treated with PMX-DHP between 2008 and 2015, four of whom were included in our previous case reports [21, 25]. The diagnosis of DM/CADM was based on Bohan and Peter’s [26, 27] and Sontheimer’s [28, 29] criteria. Patients were diagnosed with interstitial pneumonia based on computed tomography or HRCT images of the chest. The inclusion criteria for RPIPs were as follows: (1) unexplained worsening of dyspnea within 1 month; (2) evidence of hypoxemia as defined by a P/F ratio < 300 mmHg; (3) HRCT findings of newly developed ground-glass opacities and/or consolidations; (4) no evidence of pulmonary infection in bronchoalveolar lavage and sputum culture, and negative results in blood tests for other potentially infectious pathogens; and (5) exclusion of left-heart failure, pulmonary embolism, pneumothorax, and other possible causes of acute respiratory failure. Blood endotoxin levels were determined using the Endospecy test (SEIKAGAKU CORP., Tokyo, Japan). Endotoxin levels before the initial treatment and PMX-DHP therapy were within the normal range in all patients. The study protocol was approved by the Human Ethics Review Committee of Kumamoto University Hospital. For inclusion in this study, written informed consent was obtained from each patient. If the patients do not have the capacity to consent, the consent was obtained from the family member.

### Scoring of HRCT findings

The HRCT findings were graded using a classification method reported by Ichikado et al.; this method uses the following 6-point scale: (1) normal attenuation, (2) ground-glass attenuation (GGA) without traction bronchiectasis, (3) airspace consolidation without traction bronchiectasis, (4) GGA with traction bronchiectasis, (5) airspace consolidation with traction bronchiectasis, or (6) honeycombing [30]. Each lung was divided into upper, middle, and lower areas to yield a total of six zones, each of which was evaluated separately. The upper lung zone was defined as the area above the level of the tracheal carina, the lower zone was defined as the area below the level of the inferior pulmonary vein, and the middle zone was defined as the area between the upper and lower zones. In each zone, the extent of involvement of each finding was assessed visually and estimated to the nearest 10% of parenchymal involvement. The overall percentage of involvement of one type of abnormal finding in all six lung zones was obtained by averaging the corresponding values from all zones. Each abnormality score was calculated by multiplying the extent of involvement by each grading score, and the total HRCT score was calculated by summing up all of the grading scores. Two observers reviewed the HRCT findings. If the observers’ scores differed, a consensus was reached after discussion.

### PMX-DHP therapy

PMX-DHP therapy using a polymyxin B-immobilized fiber column was performed using conventional equipment for hemoperfusion and a circuit for hemodialysis, as described in our previous reports [21, 25]. For venous access, a double-lumen catheter was inserted into the femoral vein using the Seldinger technique. Direct hemoperfusion was performed at a flow rate of 80–100 mL/min for 4 h per day. PMX-DHP therapy was performed once daily on two successive days. Nafamostat mesylate (Torii Pharmaceutical Co. Ltd., Tokyo, Japan) was used as an anticoagulant. Conventional treatments, including the systemic administration of high-dose corticosteroids, empirical antibiotics, and/or immunosuppressive agents such as cyclophosphamide, tacrolimus, and cyclosporine, were initiated before PMX-DHP therapy. All patients received steroid pulse therapy (methylprednisolone 1000 mg/day for three consecutive days), followed by tapering doses of prednisolone with or without a cyclophosphamide pulse (500 mg/m^2^ every 3–4 weeks) or cyclosporine (2–3 mg/kg/day followed by adjustment to trough levels of 100–150 ng/ml), or both. Tacrolimus (0.075 mg/kg/day followed by adjustment to trough levels of 5–10 ng/ml), instead of cyclosporine, was also added to the regimen. The treatment of patients requiring ventilator management was based on a lung-protective strategy, according to previous reports [31, 32].

### Clinical and laboratory evaluations

Demographic data and the results of blood tests including a hemogram and the analysis of LDH, creatine kinase, C-reactive protein, KL-6, Surfactant protein-D (SP-D), and ferritin levels were examined before and after PMX-DHP. Serum levels of HMGB-1 were measured using an enzyme-linked immunosorbent assay (Shino-Test Corp., Tokyo, Japan). Respiratory parameters such as P/F ratio and 90-day mortality were also recorded. Anti-MDA-5 and anti-ARS antibodies were examined using commercially available kits (MESACUP™; BML, Nagoya, Japan). The enrolled patients were divided into the survivor and non-survivor groups at 90 days after PMX-DHP therapy to identify differences in clinical and laboratory data.

### Statistical analysis

Continuous variables were expressed as median values, and differences between the survivor and non-survivor groups and anti-MDA-5 antibody positive and negative groups were compared using the Mann–Whitney *U* test and Fisher’s exact test. A *p* value <0.05 was considered to indicate statistical significance. The correlations between serum ferritin levels and other clinical parameters were analyzed using Spearman’s rank correlation coefficient. Comparisons of the changes in neutrophil and platelet counts, serum LDH and HMGB-1 levels, and P/F ratio over the course of PMX-DHP therapy were analyzed by repeated-measures analysis of variance (ANOVA). Survival curves were calculated using the Kaplan–Meier method and compared among groups using the log-rank test. All statistical analyses were performed using the Statistical Package for the Social Sciences (SPSS Version 23.0; IBM Corp., Armonk, MY, USA).

## Results

### Baseline characteristics

The baseline characteristics of the patients are shown in Table 1. The survivor group contained five patients and the non-survivor group contained nine patients (median ages: 58 and 64 years old, respectively). Women outnumbered men in both groups. We detected anti-MDA-5 antibody in 10 patients, anti-threonyl (PL-7) antibody in two patients, and anti-glycyl (EJ) antibody in one patient. No autoantibodies were detected in the remaining patient. All patients were treated with high-dose corticosteroid and cyclophosphamide pulse therapy and cyclosporine or tacrolimus administration. The median intervals between corticosteroid pulse therapy and PMX-DHP were 5 days in the survivor group and 6 days in the non-survivor group.

**Table 1:** Characteristics of patients before PMX-DHP therapy

	Survivors	Non-survivors	*p* value
Case No.	5	9	
Age	64 (36–75)	58 (44–77)	0.546
Sex (Male/Female)	1/4	2/7	1.000
BMI	22.1 (20.5–24.1)	22 (20.6–28.6)	0.548
Smoking status (Never/Ex/Current)	4/1/0	7/0/2	1.000
Days from steroid pulse therapy to PMX-DHP	5 (1–11)	6 (1–48)	0.584
Anti-ARS Ab (PL-7/EJ) Anti-MDA-5 Ab	3 (2/1) 1	0 9	0.028* 0.005*
IPPV	1	4	0.580
Steroid pulse therapy	5	9	1.000
Immunosuppressive agents			
Cyclosporine	4	8	1.000
Tacrolimus	1	5	0.301
Cyclophosphamide pulse	5	9	1.000

Dates are expressed as group median values or numbers of patients

*BMI* body mass index, *PMX-DHP* direct hemoperfusion therapy using a polymyxin B-immobilized fiber column, *ARS* aminoacyl-tRNA synthetase, *PL-7*, threonyl; EJ, glycyl; *MDA-5* melanoma differentiation-associated gene 5, *IPPV* invasive positive pressure ventilation

* *p* value <0.05

### Comparison of clinical parameters between the survivor and non-survivor groups

The clinical parameters before PMX-DHP are presented in Table 2. P/F ratio was significantly higher in the survivor group than in the non-survivor group (242 versus 138.6, *p* = 0.042). The baseline APACHE II scores and HRCT scores did not differ between the groups. Serum LDH and ferritin levels were significantly lower in the survivor group than in the non-survivor group (332 versus 468 U/L, *p* = 0.014 and 180.6 versus 1260.3 ng/mL, *p* = 0.011, respectively). Serum SP-D levels and platelet counts were significantly higher in the survivor group than in the non-survivor group (150.1 versus 69.9 ng/mL, *p* = 0.039 and 31.8 × 10^4^/μL versus 21.9 × 10^4^/μL, *p* = 0.003, respectively). The platelet counts were lower among non-survivors, and only one subject in this group exhibited accompanying disseminated intravascular coagulation (DIC), while three patients exhibited accompanying thrombocytosis. None of the survivors exhibited accompanying DIC and/or thrombocytosis (data not shown).

**Table 2:** Laboratory findings of patients before PMX-DHP therapy

	Survivors (*n* = 5)	Non-survivors (*n* = 9)	*p* value
P/F ratio	242 (147.6–262.5)	138.6 (48.6–242.7)	0.042*
APACHE II	12 (9–15)	13 (11–17)	0.139
HRCT score, %	247.8 (191.8–361.2)	231.4 (177.0–309.6)	0.463
WBC, /μL	11,100 (10600–19,400)	10,000 (3300–15,600)	0.124
Neut, /μL	10,038 (7825–17,188)	9016 (2670–13,603)	0.257
Lym, /μL	1707 (214–2775)	530 (0–1310)	0.257
Plt (× 10^4^), μL	31.8 (29.7–40.7)	21.9 (9.4–26.8)	0.003*
LDH, U/L	332 (171–462)	468 (343–1104)	0.014*
CK, U/L	69 (14–335)	65 (11–514)	0.689
CRP, mg/dL	4.08 (0.14–13)	0.56 (0.13–4.22)	0.386
KL-6, U/mL	906 (390–2725)	2024 (403–4730)	0.125
SP-D, ng/mL	150.1 (116.6–339.9)	69.9 (15–341)	0.039*
Ferritin, ng/mL	180.6 (13–701.3)	1260.3 (600.8–4362)	0.011*
HMGB-1, ng/mL	9.6 (3.6–37.4)	13.2 (2.7–30.7)	0.942

Dates are expressed as group median values. The *p* values refer to comparisons between the survivors and non-survivors groups. *: *p* value <0.05

*P/F*, arterial partial pressure of oxygen/fraction of inspired oxygen; *WBC* white blood cells, *Neut* neutrophils, *Lym* lymphocytes, *Plt* platelets, *LDH* lactate dehydrogenase, *CK* creatine kinase, *CRP* C-reactive protein, *KL-6* Krebs von den Lungen-6, *SP-D* Surfactant protein-D, *HMGB-1* High-mobility group box protein 1

### Correlations between serum ferritin levels and other clinical parameters before PMX-DHP therapy

**Fig. 1:**
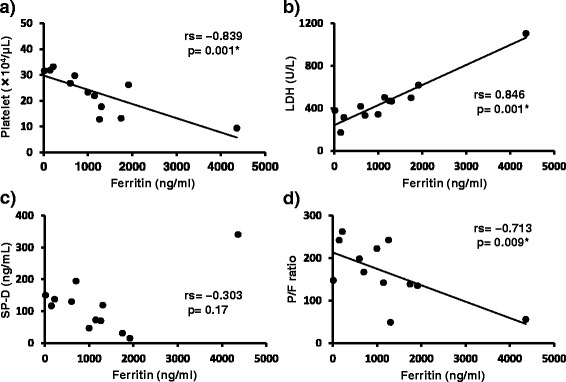
Correlations between serum ferritin levels and other clinical parameters before PMX-DHP. **a**: Platelet counts were negatively correlated with serum ferritin levels (*r*_*s*_ = −0.839, p = 0.001). **b**: Serum LDH levels were positively correlated with serum ferritin levels (*r*_*s*_ = 0.864, *p* = 0.001). **c**: There was no significant correlation between serum ferritin and SP-D levels. **d**: The P/F ratios were negatively correlated with serum ferritin levels (*r*_*s*_ = −0713, *p* = 0.009). *: *p* value <0.05

Serum ferritin level is known to predict disease activity and prognosis in patients with DM and RPIPs [33, 34]. Serum ferritin levels had a significant negative correlation with the platelet counts in peripheral blood samples and P/F ratio (Fig. 1a and d). Serum LDH levels had a significant positive correlation with serum ferritin levels, but there was no significant correlation between serum ferritin and SP-D levels (Fig. 1b and c).

### Changes in clinical parameters before and after PMX-DHP therapy

**Fig. 2:**
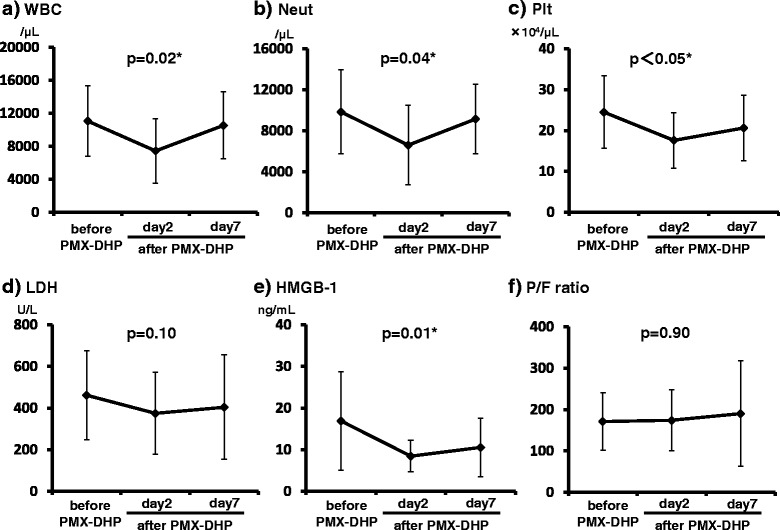
Changes in clinical parameters before and after PMX-DHP therapy. **a**, **b**: White blood cell and neutrophil counts were not significantly different before and after PMX-DHP therapy. **c**, **e**: After PMX-DHP, the platelet counts in peripheral blood samples and serum HMGB-1 levels were significantly lower than before PMX-DHP therapy. **d**: Serum LDH levels were not significantly different before and after PMX-DHP. **f**: The P/F ratio tended to improve after PMX-DHP, but there were no significant differences between P/F ratio before and after PMX-DHP therapy. *: *p* value <0.05

**Fig. 3:**
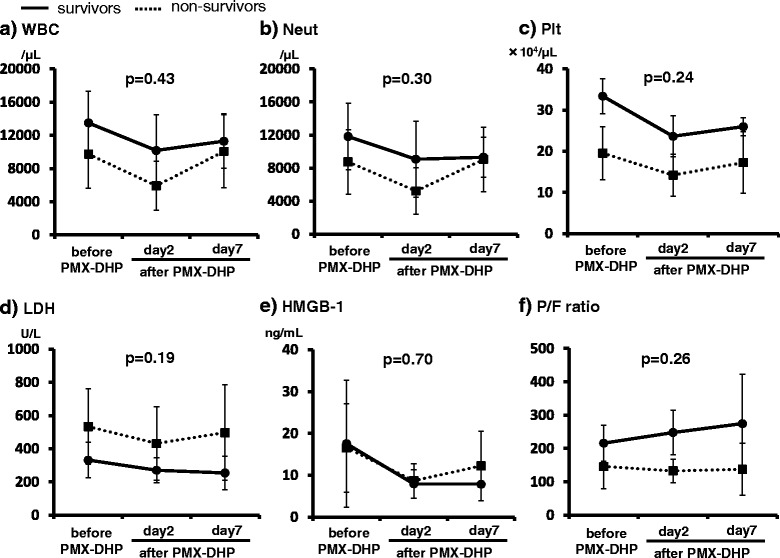
Clinical parameters differences before and after PMX-DHP in the survivor and non-survivor groups. **a**: White blood cell counts, **b**: neutrophil counts, **c**: platelet counts, **d**: serum LDH levels, **e**: serum HMGB-1 levels, **f**: and P/F ratio in the survivor (solid line and closed circles) and non-survivor (dotted line and closed squares) groups after PMX-DHP therapy. In the survivor group, serum LDH levels, serum HMGB-1 levels, and WBC, neutrophil, and platelet counts tended to decrease after PMX-DHP therapy, but there were no significant differences between the survivor and non-survivor groups

Figure 2 shows the changes in clinical parameters before and after PMX-DHP. After PMX-DHP, the platelet counts in peripheral blood samples and serum HMGB-1 level were significantly decreased. The white blood cell and neutrophil counts were significantly different before and after PMX-DHP. P/F ratio tended to improve after PMX-DHP, but there was no significant difference in P/F ratio before and after PMX-DHP. The changes in clinical parameters before and after PMX-DHP treatment showed no significant differences between the survivor and non-survivor groups (Fig. 3).

### Prognosis

**Fig. 4:**
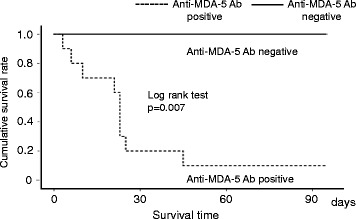
Kaplan–Meier comparison of survival curves in the anti-MDA-5 antibody positive and negative groups. There was a significant difference in 90-day survival between the anti-MDA-5 positive group (dotted line) and negative group (solid line) (*p* = 0.007)

The survival rate at 90 days after PMX-DHP was 35.7% (5/14). All patients positive for anti-ARS antibodies and one of ten patients positive for anti-MDA-5 antibody survived (Table 1). Four of five patients treated with invasive positive pressure ventilation (IPPV) died. The one survivor treated with IPPV was positive for an anti-ARS antibody. The cause of death in all non-survivors was the exacerbation of interstitial pneumonia. A Kaplan–Meier comparison of survival curves showed that the 90-day survival rate was better in anti-MDA-5 antibody negative patients than in anti-MDA-5 antibody positive patients (Fig. 4).

### Comparison of clinical parameters between the anti-MDA-5 antibody positive and negative groups

In the anti-MDA-5 antibody positive group, the platelet counts in peripheral blood samples and P/F ratio were significantly lower (*p* = 0.007, 0.024) and serum LDH levels were significantly higher (*p* = 0.024) than in the anti-MDA-5 antibody negative group (Table 3). There was no significant difference in serum ferritin levels between the two groups.

**Table 3:** Laboratory findings of patients before PMX-DHP in anti-MDA-5 Ab positive and negative groups

	Positive (*n* = 10)	Negative (*n* = 4)	*p* value
P/F ratio	140.3 (48.6–242.7)	251 (167–262.5)	0.024*
APACHE II	13 (11–17)	11 (9–15)	0.175
HRCT score, %	233.9 (177.0–309.6)	238.7 (191.8–361.2)	0.777
WBC, /μL	10,300 (3300–15,600)	13,200 (11100–19,400)	0.065
Neut, /μL	9527 (2670–13,603)	12,032 (7825–17,188)	0.258
Lym, /μL	510 (0–1310)	1774 (214–2775)	0.120
Plt (× 10^4^), μL	22.6 (9.4–31.6)	32.5 (29.7–40.7)	0.007*
LDH, U/L	466 (343–1104)	323 (171–462)	0.024*
CRP, mg/dL	0.64 (0.13–4.52)	2.17 (0.14–13)	0.888
KL-6, U/mL	1849 (403–4730)	1168 (390–2725)	0.258
SP-D, ng/mL	71.3 (15–341)	166.1 (116.6–339.9)	0.090
Ferritin, ng/mL	1260.3(13–4362)	215.9 (145.3–701.3)	0.192
HMGB-1, ng/mL	14.6 (2.7–37.4)	8.4 (3.6–30)	0.374

Dates are expressed as group median values. The p values refer to comparisons between the survivors and non-survivors groups. *: *p* value <0.05

P/F, arterial partial pressure of oxygen/ fraction of inspired oxygen; WBC, white blood cells; Neut, neutrophils; Lym, lymphocytes; Plt, platelets; LDH, lactate dehydrogenase; CK, creatine kinase; CRP, C-reactive protein; KL-6, Krebs von den Lungen-6; SP-D, Surfactant protein-D; HMGB-1, High-mobility group box protein 1; PMX-DHP, direct hemoperfusion therapy using a polymyxin B-immobilized fiber column; anti-MDA-5 Ab, anti-melanoma differentiation-associated gene 5 antibody

## Discussion

In this study, we retrospectively analyzed the clinical outcome of patients with CADM-associated RPIPs treated with PMX-DHP in addition to conventional therapies. A higher P/F ratio and serum SP-D levels, lower serum LDH and ferritin levels, positivity for anti-ARS antibodies, and negativity for anti-MDA-5 antibody were predictive of favorable outcomes. Furthermore, the survivor group exhibited higher platelet counts, which were within the normal range in almost all patients. Although PMX-DHP therapy previously demonstrated efficacy for the treatment of acute exacerbations of IPF [12, 15, 16], the mortality rate of anti-MDA-5 antibody positive patients with CADM-associated RPIPs remained poor despite additional PMX-DHP therapy.

CADM-associated RPIPs with acute respiratory failure are refractory to various intensive therapies, including respiratory support and combination therapies with high-dose corticosteroids and immunosuppressive agents such as cyclophosphamide, cyclosporine, and tacrolimus. The poor outcomes of patients with these conditions have prompted the development of new therapeutic approaches. In Japan, PMX-DHP has been used to remove endotoxin from the blood of patients with severe sepsis for the past two decades. Recently, PMX-DHP has been applied to treat non-septic ARDS and RPIPs in acute exacerbations of IPF, CADM-associated RPIPs, and fatal drug-induced interstitial pneumonias in which endotoxin was not detected in the blood [12–16, 21–24]. The mechanism by which PMX-DHP therapy affects RPIPs has not been fully elucidated. However, several reports have discussed the mechanism of the PMX-DHP. Abe et al. [18, 19] showed that PMX-DHP treatment eliminated activated neutrophils and humoral factors, including matrix metalloproteinase-9 and HMGB-1, which are relevant to an improved P/F ratio, from the blood circulation in patients with acute exacerbations of IPF. Furthermore, Oishi et al. reported prominent decreases in the serum levels of vascular endothelial growth factor (VEGF) and IL-12 after PMX-DHP therapy, and observed a correlation between improved oxygenation after PMX-DHP therapy and the quantity of VEGF eluted from PMX-fiber cartridges [35].

The presence of the anti-MDA-5 antibody in patients with CADM-associated RPIPs is known to indicate a poor prognosis: its titer reflects disease activity and responses to treatment [3–5]. In this study, all of the nine non-survivors tested positive for anti-MDA-5 antibody, suggesting that anti-MDA-5 antibody is an important prognostic marker in patients with CADM. Only one anti-MDA-5 antibody positive patient survived following treatment with conventional intensive pharmacotherapy and PMX-DHP. In contrast, interstitial lung disease (ILD) with positivity for anti-ARS antibodies is usually associated with a chronic course and a good response to corticosteroid therapy [36]. In this study, however, three patients positive for anti-ARS antibodies suffered acute progressive respiratory failure despite treatment with a combination of high-dose corticosteroids and immunosuppressive agents, and required PMX-DHP as an additional therapy to save their lives (Fig. 4). Further studies are needed to save the lives of more patients with CADM-associated RPIPs.

In this study, higher serum ferritin levels were associated with poor prognosis. Serum ferritin levels are correlated with the activity of CADM-associated ILD [33, 34]. Ferritin is an intracellular protein responsible for iron storage that is mainly secreted by activated macrophages. Higher serum ferritin levels may reflect aberrant ferritin production by activated macrophages in patients with RPIPs. Interestingly, in this study, the only patient who survived of 10 patients positive for anti-MDA-5 antibody exhibited a ferritin level within the normal range and lower than all other patients. We found that serum ferritin levels were significantly correlated with P/F ratio, platelet counts, and serum LDH levels, all of which were also markers for survival.

Our results showed that an increased platelet count in the peripheral blood was a good prognostic factor. To date, no studies have reported an associated between platelet count and outcome in patients with DM-associated RPIPs. A retrospective analysis of thoracoscopic biopsy specimens from patients in the fibroproliferative phase of ARDS indicated that megakaryocytes are present in the microvessels of the injured lung. The mortality rate was greater in patients with thrombocytopenia than in those with thrombocytosis in this study [37]. Because platelets are generated via the cytoplasmic fragmentation of megakaryocytes within the pulmonary capillary bed [37, 38], thrombocytopenia may be induced by the destruction of pulmonary microvessels in CADM-associated RPIPs.

In this study, the serum SP-D levels of non-survivors were significantly lower than those of survivors. Ikeda et al. [39] reported that the serum SP-D levels in anti-MDA-5 antibody positive group were significantly lower than those in the negative group. These lower SP-D levels in the non-survivor group may reflect poor cellularity and progressive fibrotic change.

In this study, serum HMGB-1 levels in patients with CADM-associated RPIPs increased before and significantly decreased after PMX-DHP therapy in both the survivor and non-survivor groups. Shu et al. [40] reported that the serum HMGB-1 levels of PM/DM patients with ILD were significantly higher than those of PM/DM patients without ILD, and that patients with higher HMGB-1 levels had significantly worse prognosis than those with lower HMGB-1 levels. In our study, although the serum levels of HMGB-1 were significantly decreased by PMX-DHP treatment, the levels were unrelated to the outcome. Further studies are required to determine the precise mechanism of action of PMX-DHP therapy for CADM-associated RPIPs.

This study has several limitations. First, this was a small, retrospective study, which may have been subject to various biases. Some negative or positive associations in the statistical analyses may have been due to the inadequate power afforded by the small sample size. Therefore, larger-scale prospective studies are needed to confirm our results. Second, none of the patients in this study were treated using the same protocol. In our study, all patients were treated with combination therapy involving corticosteroids, immunosuppressive therapy, and PMX-DHP therapy. The decision regarding the choice of immunosuppressive agents was made by the attending physicians, and the types and amounts of these agents and timing of their administration varied between patients. Furthermore, the initiation of PMX-DHP varied in this study. In patients with ARDS and RPIPs including acute exacerbations of IPF, early induction of PMX-DHP therapy yielded better results and had a significant impact on survival [10, 41]. The variations in therapeutic regimen may have affected the responses to therapy and outcomes observed in this study.

## Conclusion

CADM-associated RPIPs with anti-MDA-5 antibody is associated with a very poor prognosis. In CADM-associated RPIPs treated with PMX-DHP, higher P/F ratio, platelet counts in peripheral blood samples, and serum SP-D levels, lower serum LDH and ferritin levels before PMX-DHP, positivity for anti-ARS antibodies, and negativity for anti-MDA-5 antibody indicate a favorable prognosis. Further studies are needed to establish the effect of PMX-DHP therapy and develop better therapeutic management for patients with CADM-associated RPIPs.

## Abbreviations

ARDS: Acute respiratory distress syndrome
ARS: Aminoacyl-tRNA synthetase
CADM: Clinically amyopathic dermatomyositis
DIC: Disseminated intravascular coagulation
HMGB-1: High-mobility group box protein 1
HRCT: High-resolution computed tomography
ILD: Interstitial lung disease
IPF: Idiopathic pulmonary fibrosis
IPPV: Invasive positive pressure ventilation
KL-6: Krebs von den Lungen-6
LDH: Lactate dehydrogenase
MDA-5: Melanoma differentiation-associated gene 5
MMP-9: Matrix metalloproteinase-9
P/F: Arterial partial pressure of oxygen/fraction of inspired oxygen
PM: Polymyositis
PMX-DHP: Direct hemoperfusion therapy using a polymyxin B-immobilized fiber column
RPIP: Rapidly progressive interstitial pneumonia
SP-D: Surfactant protein-D
